# Optic Neuropathy Caused by a Perineural Sarcoid Lesion

**DOI:** 10.7759/cureus.21966

**Published:** 2022-02-06

**Authors:** Yasuhiro Takahashi, Shinjiro Kono, Aric Vaidya, Hirohiko Kakizaki

**Affiliations:** 1 Oculoplastic, Orbital and Lacrimal Surgery, Aichi Medical University Hospital, Nagakute, JPN; 2 Oculoplastic, Orbital and Lacrimal Surgery, Kirtipur Eye Hospital, Kathmandu, NPL

**Keywords:** vision loss, steroid, optic neuropathy, perineural mass, sarcoidosis

## Abstract

A 74-year-old woman had a six-month history of decreased vision in the left eye. On the first examination, her left best-corrected visual acuity was 0.02, and Goldmann visual field test revealed a central scotoma in the left eye. Magnetic resonance imaging demonstrated lesions around the optic nerve on both sides and enlargement of the lacrimal gland and superior rectus/levator palpebrae superioris muscles on both sides and the medial and inferior recti muscles on the left side. Systemic computed tomography revealed bilaterally enlarged mediastinal and supraclavicular lymph nodes. The blood test results included an elevated soluble interleukin-2 receptor. Pathological examination of the specimens harvested from the lacrimal gland on both sides, left levator palpebrae superioris muscle, and the lesion around the optic nerve on the left side showed lymphocytic infiltration with noncaseating epithelioid granuloma. After the biopsy, the patient underwent two cycles of steroid pulse therapy, followed by oral prednisolone. Although the lesions were reduced after steroid treatment, the left vision did not recover.

## Introduction

Sarcoidosis involves the ocular and periocular tissues in 10%-60% of cases [1-4]. The most common manifestation of ocular sarcoidosis is uveitis, while extraocular lesions are relatively rare [1-6]. Involvement of the lacrimal gland is most frequent, followed by the orbit, eyelid, and lacrimal sac [1], while the optic nerve is rarely involved [2].

The neuro-ophthalmic sarcoidosis is the second frequent type in neurosarcoidosis and is found in 1%-5% of cases with sarcoidosis [3,7,8]. Most cases show optic perineuritis, while other manifestations including optic nerve compression by an orbital mass, optic nerve granulomas, and papilloedema by elevated intracranial pressure are rare [7,9-11]. We report a case of optic neuropathy secondary to a perineural sarcoid mass.

## Case presentation

A 74-year-old woman complained of decreased vision six months before referral to us. The patient had followed up at an eye clinic for cataract in both eyes, but she searched another clinic for further examination of gradually decreased vision on her own accord. Multiple orbital masses and lung and breast lesions were detected at the second clinic. She did not have history of any systemic disease or family history.

**Figure 1:**
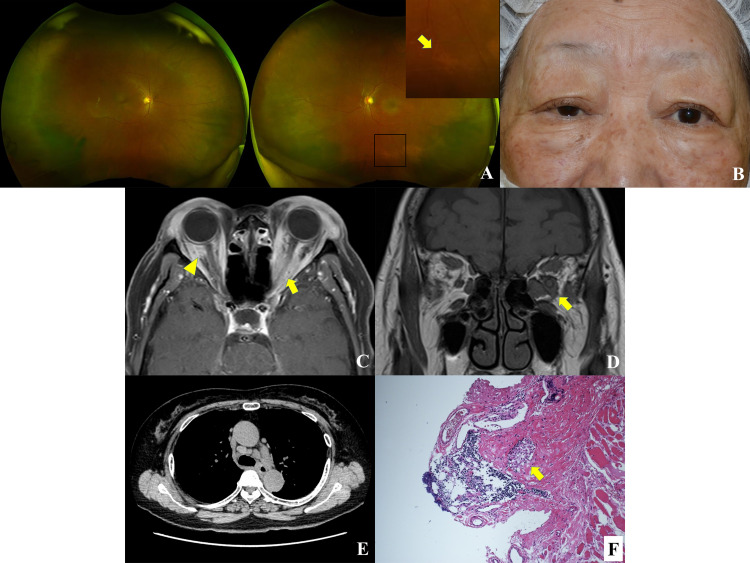
Case presentation. A. A left fundus photo showing a pale disc and mild retinal phlebitis (arrow). B. A face photo showing proptosis on the left side and ptosis on the right side. C, D. An enhanced T1-weighted axial (C) and T1-weighted coronal magnetic resonance images (MRI) (D) showing lesions around the optic nerve on both sides (arrowhead and arrow) with the “tram-track” sign on the left side (arrow) and enlargement of the bilateral lacrimal glands, bilateral superior rectus/levator palpebrae superioris muscles, and left medial and inferior recti muscles. E. An axial chest computed tomographic images showing enlarged mediastinal lymph nodes. F. Pathological examination showing lymphocytic infiltration with noncaseating epithelioid granuloma (arrow; hematoxylin and eosin staining; magnification, ×100).

On the first examination, her best-corrected visual acuity was 1.0 in the right eye and 0.02 in the left eye. Intraocular pressure was 13 mmHg in both eyes. Slit-lamp examination did not show cells in the anterior chamber, iris nodule, or peripheral angle synechiae. Funduscopic examination revealed a pale optic disc and mild retinal phlebitis in the left eye (Figure 1A). Relative afferent pupillary defect (RAPD) was positive in the left eye. Goldmann visual field test revealed a central scotoma in the left eye. Abduction and supraduction were restricted in the left eye. The left eye had 1.5 mm proptosis as compared to the right eye (Figure 1B). The lacrimal gland was palpable in both sides. The right upper eyelid was ptotic, and marginal reflex distance 1 was 0.5 mm on the right side and 3.5 mm on the left side (Figure 1B). Magnetic resonance imaging (MRI) demonstrated lesions around the optic nerve on both sides and enlargement of the lacrimal gland and superior rectus/levator palpebrae superioris muscles on both sides and the left medial and inferior recti muscles (Figures 1C, 1D). The lesions showed isointensity to muscle on T1- and T2-weighted MRI images. Enhanced T1-weighted axial MRI showed the “tram-track” sign on the left side (Figure 1C). Systemic computed tomography revealed a mass in the right breast and bilaterally enlarged mediastinal and supraclavicular lymph nodes (Figure 1E). The blood test results included an elevated soluble interleukin-2 receptor (1,153 U/mL; normal range, 122-496 U/mL) and antinuclear antibody (1:40; normal range, < 1:40). Thyroid autoantibodies, rheumatoid factor, Sjögren syndrome (SS)-A and SS-B antibodies, immunoglobulin G4, anti-DNA antibody, proteinase 3- and myeloperoxidase-antineutrophil cytoplasmic antibodies, and angiotensin-converting enzyme were within the normal ranges. The patient was consulted with a breast surgeon, but the doctor judged no necessity for further examination of the breast lesion.

An incisional biopsy was performed under general anesthesia by two of the authors (YT and SK). The lesions in the lacrimal gland on both sides and levator palpebrae superioris muscle on the left side were partially excised via an eyelid crease approach. Next, the lesion around the left optic nerve was biopsied via a transconjunctival approach of the lower eyelid. After the surgery, the patient underwent two cycles of steroid pulse therapy. Pathological examination of the harvested specimens showed lymphocytic infiltration with noncaseating epithelioid granuloma (Figure 1F). A pulmonologist diagnosed it as a pulmonary sarcoidosis based on the findings of enlarged mediastinal lymph nodes and elevated serum interleukin-2 receptor, while the cardiologist and dermatologist ruled out the presence of cardiac and cutaneous sarcoidosis, respectively.

After completing the steroid pulse therapy, the patient started taking oral prednisolone and tapered it from 30 mg/day over a period of six months. Following steroid treatment, although the lesions were reduced in size, the left vision did not recover. Ptosis on the left side developed after the biopsy, and margin reflex distance 1 was -2.0 mm on the left side.

This case report adheres to the tenets of the Declaration of Helsinki as amended in 2008. Written informed consent for publication of patient record and identifiable patient face photos was obtained from the patient.

## Discussion

We present a rare case of optic neuropathy secondary to a perineural sarcoid mass. Mixed results from previous studies demonstrated only four cases of optic nerve involvement and the other two cases with positive RAPD among 137 cases with orbital sarcoidosis [1,2,4,6]. Additionally, there had been a few case reports on orbital sarcoidosis with optic nerve involvement [7,10,11].

The histopathological findings of the specimens harvested from the orbits corresponded to the histological diagnostic criteria of sarcoidosis proposed by the Japan Society of Sarcoidosis and Other Granulomatous Disorders in 2015 [12]. Furthermore, bilateral enlargement of the mediastinal and supraclavicular lymph nodes and elevated soluble interleukin-2 receptor fulfilled the clinical diagnostic criteria of sarcoidosis [12].

Sarcoidosis is considered a “great mimicker”. A previous report showed a case of a sarcoid mass around the optic nerve presenting with “tram-track” sign, similar to optic nerve meningioma [11]. Our case had similar findings including perineural mass and the “tram-track” sign on enhanced axial MRI. However, bilateral lesions and other accompanying orbital lesions are rare in cases with optic nerve sheath meningioma [13]. Other differential diagnoses include metastatic tumors, leukemia, lymphoma, and other orbital inflammatory diseases [3,14]. However, it may be possible to differentiate these diseases, based on the results of blood tests and systemic imaging studies.

Treatment modalities for orbital sarcoidosis include intralesional, oral, and intravenous steroids, methotrexate, cyclosporine, cyclophosphamide, mycophenolate, interferon, azathioprine, hydroxychloroquine, and surgical debulking [1-3,6,14]. However, intravenous steroids, which were given in the present case, may be a better option for cases with optic nerve involvement [14].

The vision recovered after steroid treatment in previously reported cases with optic nerve involvement [7,10]. However, in our case, there was no recovery of left vision even after steroid treatment. Those previously reported cases had optic nerve edema before starting steroid treatment, while in our case, the optic nerve disc was already pale. This may be a cause of the different clinical course between the present and the previously reported cases.

## Conclusions

In conclusion, we report a rare case of optic neuropathy caused by a perineural sarcoid mass. The findings of our case fulfilled both the histological and clinical diagnostic criteria of sarcoidosis. Preoperative blood tests and orbital and systemic imaging studies are essential for the definite diagnosis of neuro-ophthalmologic sarcoidosis.
